# Supplementary material for: Clinical Features, Gene Alterations, and Outcomes in Prefibrotic and Overt Primary and Secondary Myelofibrotic Patients
Source: Cancers (Basel). 2022 Sep 16;14(18):4485. doi: 10.3390/cancers14184485 (PMC9496754; doi:10.3390/cancers14184485)
Supplement: Supplementary file 1 [file cancers-14-04485-s001.zip › cancers-1879733-supplementary.pdf]

Supplementary Data

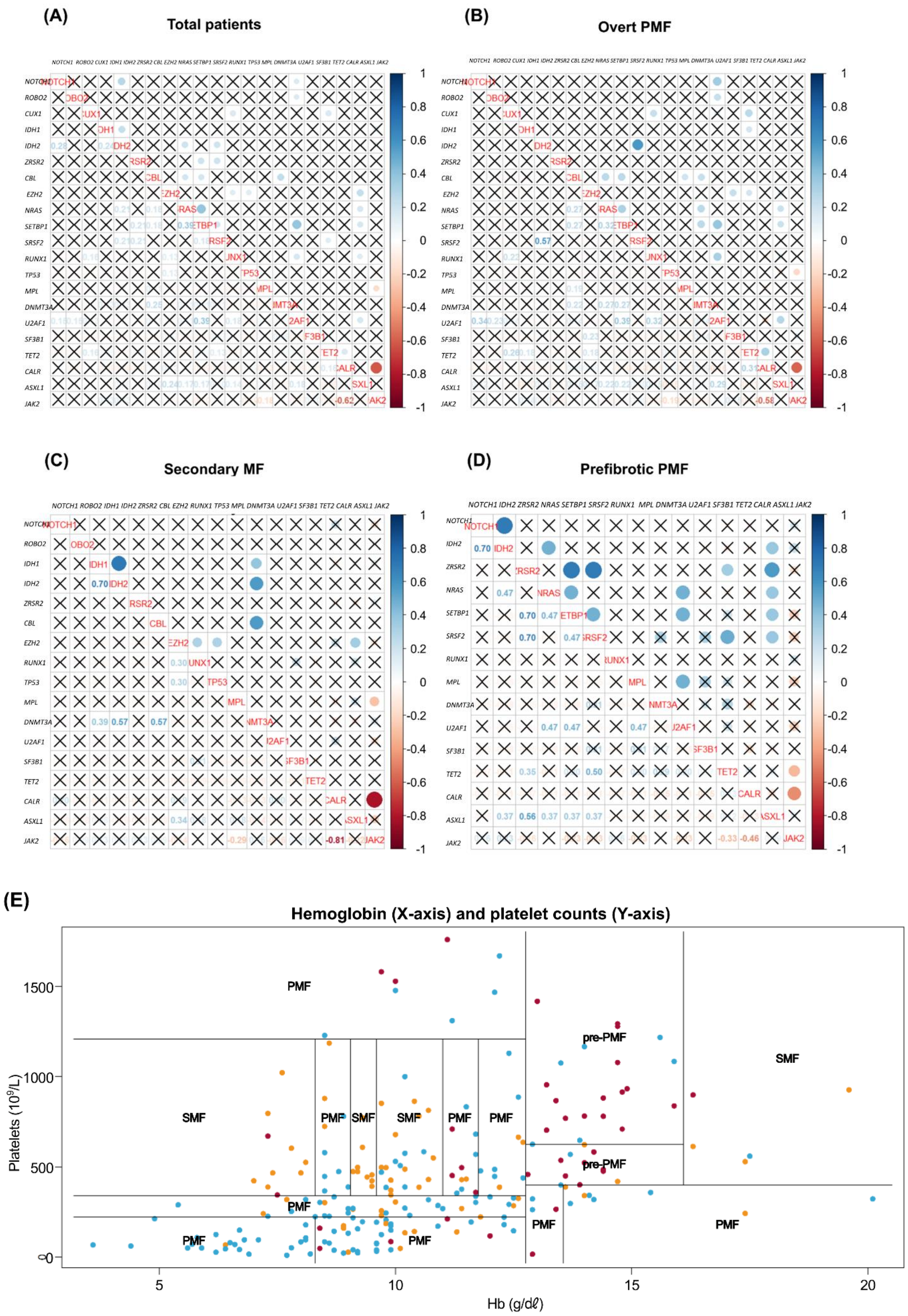

Supplementary Figure S1. Correlation between mutations in (A) overall cohort, (B) primary myelofibrosis (PMF), (C) secondary myelofibrosis (SMF), and (D) prefibrotic myelofibrosis (pre-PMF). X stands for p-value over 0.05; Numbers are correlation coefficient.

(E) Partition of PMF, SMF, and pre-PMF by the decision tree method according to hemoglobin level (X-axis) and platelet counts (Y-axis).

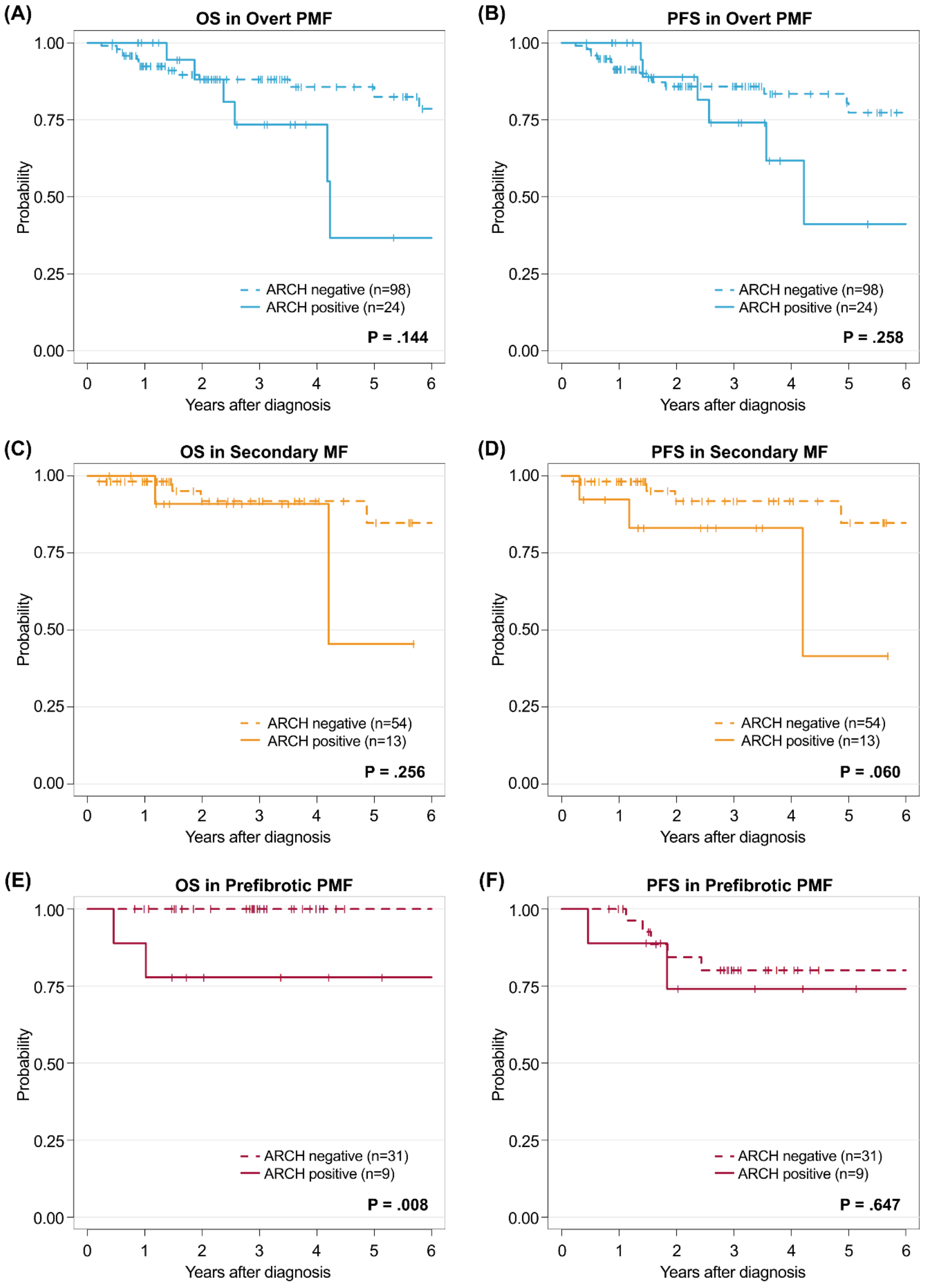

Supplementary Figure S2. Estimation of overall survival (OS) in (A) primary myelofibrosis (PMF), (C) secondary myelofibrosis (SMF), and (E) prefibrotic myelofibrosis (pre-PMF), and progression-free survival (PFS) in (B) PMF, (D) SMF, and (F) pre-PMF following CHIP of the high-risk progression group.

Supplementary Table S1. List of target genes in the NGS panel.

Target genes	Interval	Number of regions	Size (base pairs)
ABCA12	chr2:215797358-216002931	54	7929
ABL1	chr9:133589707-133761070	12	3529
ASXL1	chr20:30946579-31025141	17	4720
ATM	chr11:108098352-108236235	62	9171
ATRX	chrX:76763829-77041487	36	7543
ATXN7L1	chr7:105248299-105517004	15	3132
BCOR	chrX:39909169-39937182	15	5348
BRAF	chr7:140426294-140624503	21	2379
BRCC3	chrX:154299803-154348425	11	1021
CALR	chr19:13049494-13054795	10	1294
CBL	chr11:119077128-119170491	16	2721
CBLB	chr3:105377814-105588232	20	3079
CD101	chr1:117544440-117576723	9	3066
CEBPA	chr19:33792244-33793425	1	1182
CREBBP	chr16:3777719-3929917	31	7368
CSF1R	chr5:149433632-149465990	22	3003
CSF3R	chr1:36931697-36945097	16	2698
CTCF	chr16:67644736-67671775	10	2184
CUX1	chr7:101459311-101926382	34	5433
DNMT1	chr19:10244343-10311559	43	5292
DNMT3A	chr2:25457148-25536853	25	2888
EGFR	chr7:55086971-55273310	31	4084
EP300	chr22:41489009-41574960	31	7245
ERG	chr21:39739557-39947624	12	1764
ETV6	chr12:11803062-12044535	10	1443
EZH2	chr7:148504738-148544390	21	2456
FBXW7	chr4:153244033-153332955	14	2618
FLT3	chr13:28578189-28674647	25	3004
GATA1	chrX:48649517-48652675	5	1346
GATA2	chr3:128199862-128205874	5	1443
GNAS	chr20:57415162-57485884	17	4096
HIPK2	chr7:139257673-139477422	16	3708
IDH1	chr2:209101803-209116275	8	1248
IDH2	chr15:90627498-90645622	11	1359
INVS	chr9:102866804-103062956	17	3395
IRF1	chr5:131819643-131825170	9	978
JAK2	chr9:5021988-5126791	23	3399
KDM2B	chr12:121867919-122018816	29	4276
KDM6A	chrX:44732798-44970656	31	4470
KIT	chr4:55524182-55604723	21	2931
KMT2A	chr11:118307228-118392887	38	12082
KMT2D	chr12:49415563-49449107	55	16662
KRAS	chr12:25362729-25398318	6	708
LAMB4	chr7:107664484-107763609	35	5507
MECOM	chr3:168802697-169381160	20	3816
MET	chr7:116335811-116436178	21	4359
MLL3	chr7:151833917-152132871	62	15030
MLL5	chr7:104681400-104753780	27	5743
MN1	chr22:28146903-28196531	2	3963
MPL	chr1:43803520-43818443	12	1993
NCOR2	chr12:124809948-124979797	49	7734
NF1	chr17:29422226-29705949	63	9011
NLRP1	chr17:5405134-5487277	18	4493
NOTCH1	chr9:139390523-139440238	34	7668
NPM1	chr5:170814953-170837569	12	894

NRAS	chr1:115251156-115258781	4	570
NRD1	chr1:52254908-52344287	34	3696
NUP98	chr11:3692612-3803347	35	5567
OCA2	chr15:28000534-28327020	24	2573
PDGFRA	chr4:55106220-55161439	24	3450
PHF12	chr17:27233201-27278622	17	3519
PHF6	chrX:133511648-133559360	9	1207
PRPF40B	chr12:50017374-50037975	26	2682
PRPF8	chr17:1553953-1587865	42	7151
PTPN11	chr12:112856916-112942568	16	1822
RAD21	chr8:117859739-117878968	13	1896
RAD50	chr5:131893017-131978781	27	4211
RINT1	chr7:105172763-105207758	16	2417
ROBO1	chr3:78648063-79639061	34	5223
ROBO2	chr3:75986645-77695209	32	4862
RUNX1	chr21:36164432-36421196	11	1584
RUNX1T1	chr8:92972470-93115112	20	2350
SETBP1	chr18:42281312-42643663	6	4980
SF3A1	chr22:30730583-30752781	16	2382
SF3B1	chr2:198257027-198299723	27	4045
SMC1A	chrX:53407024-53449549	26	3882
SMC3	chr10:112327575-112364060	29	3654
SRSF2	chr17:74732243-74733242	2	666
STAG2	chrX:123156478-123234447	34	3861
TET1	chr10:70332096-70451571	11	6411
TET2	chr4:106111627-106197676	10	6165
TP53	chr17:7565257-7579912	14	1378
TP53BP1	chr15:43699581-43785241	31	6130
U2AF1	chr21:44513212-44527604	9	790
U2AF2	chr19:56166471-56185434	14	1541
WT1	chr11:32410604-32456891	11	1568
ZRSR2	chrX:15808619-15841365	12	1690

Supplementary Table S2. Genetic distribution of non-driver mutations in five subgroups by hierarchical clustering methods

	Cluster 1 (n = 104)	Cluster 2 (n = 44)	Cluster 3 (n = 56)	Cluster 4 (n = 11)	Cluster 5 (n = 15)	P
Myelofibrosis						.166
PMF, n (%)	53 (51.0)	25 (56.8)	35 (62.5)	4 (40.0)	5 (33.3)	
SMF, n (%)	28 (26.9)	11 (25.0)	18 (32.1)	3 (30.0)	7 (46.7)	
pre-PMF, n (%)	23 (22.1)	8 (18.2)	3 (5.4)	3 (30.0)	3 (20.0)	
JAK2, n (%)	56 (53.8)	21 (47.7)	28 (50.0)	8 (80.0)	7 (46.7)	.426
*CALR*_type, n (%)						.473

Type1/like	17 (16.3)	7 (15.9)	12 (21.4)	0 (0.0%)	3 (20.0)	
Type2/like	6 (5.8)	2 (4.5)	4 (7.1)	1 (10.0%)	3 (20.0)	
Others	2 (1.9)	0 (0.0)	2 (3.6)	0 (0.0%)	1 (6.7)	
MPL, n (%)	3 (2.9)	2 (4.5)	4 (7.1)	1 (10.0%)	0 (0.0)	.553
ASXL1, n (%)	0 (0.0)	11 (25.0)	55 (98.2)	0 (0.0%)	0 (0.0)	<.001
CBL, n (%)	0 (0.0)	5 (11.4)	0 (0.0)	0 (0.0%)	0 (0.0)	<.001
CUX1, n (%)	0 (0.0)	4 (9.1)	0 (0.0)	0 (0.0)	0 (0.0)	.002
DNMT3A, n (%)	0 (0.0)	11 (25.0)	0 (0.0)	0 (0.0)	0 (0.0)	<.001
EZH2, n (%)	0 (0.0)	0 (0.0)	5 (8.9)	0 (0.0)	0 (0.0)	.003
IDH1, n (%)	0 (0.0)	4 (9.1)	0 (0.0)	0 (0.0)	0 (0.0)	.002
IDH2, n (%)	0 (0.0)	2 (4.5)	2 (3.6)	0 (0.0)	0 (0.0)	.250
NOTCH1, n (%)	0 (0.0)	3 (6.8)	0 (0.0)	0 (0.0)	0 (0.0)	.012
NRAS, n (%)	0 (0.0)	2 (4.5)	3 (5.4)	0 (0.0)	0 (0.0)	.154
RUNX1, n (%)	0 (0.0)	6 (13.6)	2 (3.6)	0 (0.0)	0 (0.0)	.001
SETBP1, n (%)	0 (0.0)	3 (6.8)	2 (3.6)	0 (0.0)	0 (0.0)	.099
SF3B1, n (%)	0 (0.0)	2 (4.5)	5 (8.9)	10 (100.0)	0 (0.0)	<.001
SRSF2, n (%)	0 (0.0)	1 (2.3)	4 (7.1)	0 (0.0)	0 (0.0)	.053
TET2, n (%)	0 (0.0)	7 (15.9)	6 (10.7)	0 (0.0)	15 (100.0)	<.001
TP53, n (%)	0 (0.0)	7 (15.9)	1 (1.8)	0 (0.0)	0 (0.0)	<.001
U2AF1, n (%)	0 (0.0)	11 (25.0)	0 (0.0)	0 (0.0)	0 (0.0)	<.001
ZRSR2, n (%)	0 (0.0)	3 (6.8)	1 (1.8)	0 (0.0)	0 (0.0)	.064

SMF, secondary myelofibrosis; PMF, primary myelofibrosis; pre-PMF, prefibrotic, myelofibrosis; n, number; P, p-value

Supplementary Table S3. *p*-value of OS and PFS in between clusters.

	C1 vs the other clusters	C2 vs the other clusters	C3 vs the other clusters	C1 vs C2	C1 vs C3	C2 vs. C3
**Whole cohort**						
OS, *p*-value	.035	.002	.386	.002	.079	.144
PFS, *p*-value	.033	.002	.421	.002	.088	.140
**PMF**						
OS, *p*-value	.188	.163	.468	.162	.226	.492
PFS, *p*-value	.097	.084	.677	.073	.224	.335
**SMF**						
OS, *p*-value	.239	.001	.411	.008	.946	.016
PFS, *p*-value	.157	.001	.872	.008	.449	.066
**pre-PMF**						
OS, *p*-value	.107	.317	.013	.114	.006	.522
PFS, *p*-value	.458	.522	.002	.468	.005	.152
